# Abdominal Aorta Screening During Routine Transthoracic Echocardiography in Zanzibar, Tanzania: The Zanzibar Heart Survey

**DOI:** 10.5334/gh.1432

**Published:** 2025-05-30

**Authors:** Abukar Mohamed Ali, Khamis Mustafa Khamis, Ghirmay Andemichael, Muhiddin Abdi Mahmoud, Victor Aboyans, Sahrai Saeed

**Affiliations:** 1 Department of Heart Disease, Haukeland University Hospital, Bergen, Norway; 2 Department of Heart Disease, Mnazi Mmoja Referral Hospital, Zanzibar, United Republic of Tanzania; 3 WHO Liaison Officer & Public Health Advisor, Zanzibar, United Republic of Tanzania; 4 Department of Nephrology, Mnazi Mmoja Referral Hospital, Zanzibar, United Republic of Tanzania; 5 Department of Cardiology, Dupuytren University Hospital, and EpiMaCT, Inserm 1094 and IRD 270 unit, Limoges University, Limoges, France; 6 Department of Cardiology, Oslo University Hospital Ullevaal, Norway; 7 Faculty of Medicine, University of Oslo, Oslo, Norway

**Keywords:** Abdominal aortic aneurysms, Abdominal aorta screening, Echocardiography, Sub-Saharan Africa, Zanzibar

## Abstract

**Background and objectives:**

Abdominal aortic (AA) aneurysms (AAA) are often incidental findings and preceded by a long period of subclinical growth in diameter. Patients may present with life-threatening complications. Therefore, screening programmes for AAA in primary care are proposed in several European countries, and opportunistic AAA screening during echocardiography is also advocated. However, data on the interest of such an approach in the sub-Saharan African population are unknown.

**Methods:**

In 2022, a total of 189 patients with cardiac symptoms visiting the Mnazi Mmoja Referral Hospital (MMH) in Zanzibar underwent standard transthoracic echocardiography (TTE). Demographics and clinical data were recorded. AA diameter was routinely assessed in 137 patients. AA was measured by the leading-edge-to-leading-edge method from a longitudinal plane, and AAA was defined as an AA diameter of ≥3.0 cm. SPSS version 29.0 was used for data analysis. The prevalence of AAA was estimated as the number of AAA cases divided by the number of all screened subjects. Correlates of AA diameter were tested in univariate and multivariate linear regression analyses.

**Results:**

AA could be visualized in 128 (93.4%) patients (43% of men and 57% of women). The mean age was 54.4 ± 15.9 years. The mean AA diameter was 2.1 ± 0.3 cm in the entire study population and was significantly greater in men than women (2.2 ± 0.3 vs 2.1 ± 0.3 cm, p = 0.005) and in individuals aged ≥60 years than those aged <60 years (2.3 ± 0.3 vs 2.1 ± 0.3 cm, p = 0.003). The prevalence of AAA was 1.6%. In a multivariate linear regression analysis, higher age, male gender, atrial fibrillation and left ventricular (LV) mass were independent correlates of greater AA diameter, adjusted for clinic systolic blood pressure, ascending aortic diameter and LV ejection fraction (multiple *R^2^* = 0.38, *p* < 0.001).

**Conclusions:**

Abdominal aorta screening during routine TTE is feasible in Africa. Patients in Zanzibar have relatively smaller abdominal aorta diameters with a 1.6% prevalence of AAA. Abdominal aorta screening by routine echocardiography may be beneficial, provided that access to care and vascular surgery facility/expertise with appropriate follow-up is available for patients with AAA identified during screening.

## Introduction

Abdominal aortic (AA) aneurysm (AAA) is an abnormal focal dilation in any segment of the abdominal aorta (1), with a prevalence of 2–4% in general populations mostly of European descent (2,3). The aetiology is complex and multifactorial, and common risk factors are advanced age, male sex, atherosclerosis, hypertension, family history of AAA and history of aortic dissection (4,5,6,7). Additionally, some infectious diseases may be common in patients living in low-income countries. Genetic background also plays an important role in the development of AAA. AAA is often an incidental finding preceded by a long period of subclinical growth in diameter. Patients may present with life-threatening complications, with survival rates of 10–20% (8). Indeed, the diameter of an AAA is currently the only validated measure of rupture risk, which varies from 9% per year for a diameter range of 5.5–5.9 cm to 33% per year for a diameter of >7 cm (9). Women present with a ruptured AAA of average diameter 10 mm smaller than men (10). Accordingly, in some European countries, targeted screening programmes for AAA in primary care are increasingly being implemented, which is an important contribution to the timely management of AAA, supported by evidence from larger prospective multicentre research studies (11). However, similar population-wide AAA screening programmes are not implemented in African countries, particularly in the sub-Saharan region, probably because there are currently no large-scale baseline data regarding the burden of AAA for comparison. We postulated that opportunistic echocardiography-based screening programmes might be of interest for AAA screening in the sub-Saharan African population. Hence, the principal aims of this cross-sectional study, based on data derived from the Zanzibar Heart Survey, were to measure the AA diameter, identify risk factors associated with greater AA diameters and assess the burden of AAA in women and men undergoing transthoracic echocardiography (TTE) for any medical reason.

## Methods

**Figure 1:**
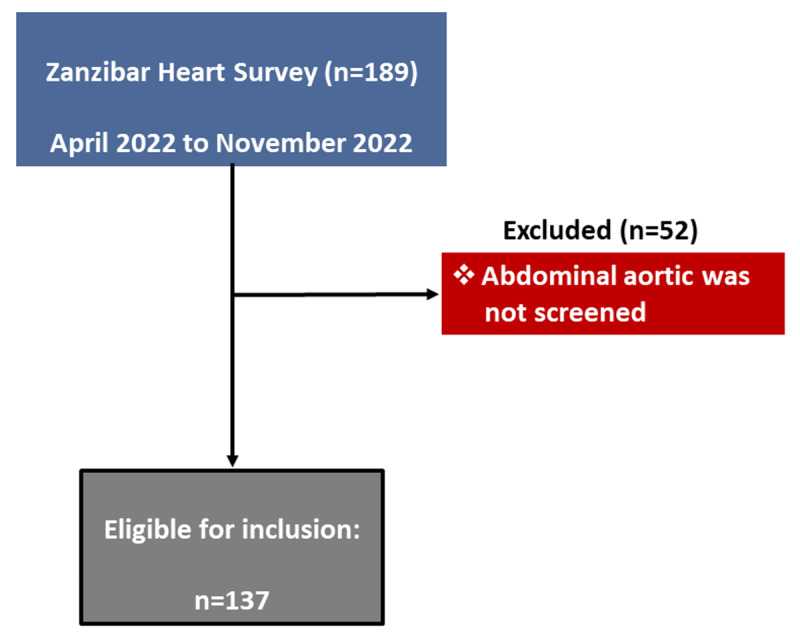
Study flow chart.

The general study design of the Zanzibar Heart Survey was recently published in detail (12). Briefly, from April 2022 to November 2022, a voluntary cardiology team from the Department of Heart Disease, Haukeland University Hospital, Bergen, Norway, was deployed to visit Zanzibar and undertake four humanitarian missions to provide clinical and echocardiographic expertise for patients presenting with cardiac symptoms. A total of 189 adult patients underwent first-ever TTE for clinical reasons during outpatient attendances at Mnazi Mmoja Referral Hospital (MMH), which formed the basis of the Zanzibar Heart Survey. Inclusion criteria were men and women aged >16 years, living in MMH – Zanzibar catchment area, with cardiac symptoms and with no previous echocardiography or heart operations. AA diameter was routinely assessed in 137 patients who were examined with TTE (Figure 1). Of these, AA could be visualized in 128/137 (93.4%) patients.

All patients underwent a thorough clinical assessment and standard blood pressure (BP) measurement by the local cardiologists prior to the echocardiographic assessment (13).

### Echocardiography

**Figure 2:**
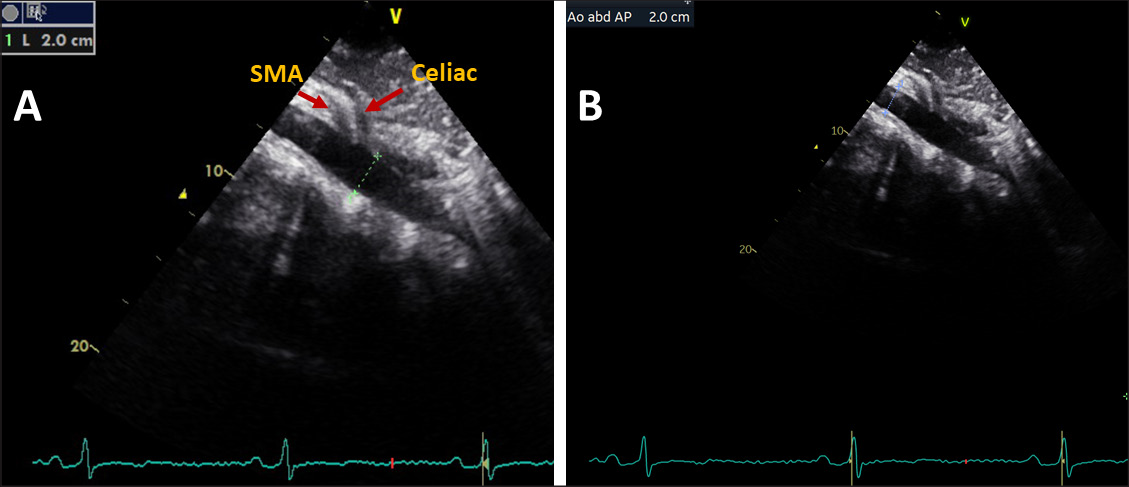
Longitudinal image of the aorta from a 29-year-old male participant. Measurements are performed above **(A)** and below **(B)**. Celiac trunk and superior mesenteric artery (SMA) branch with an anterior–posterior diameter of 2 cm.

**Figure 3:**
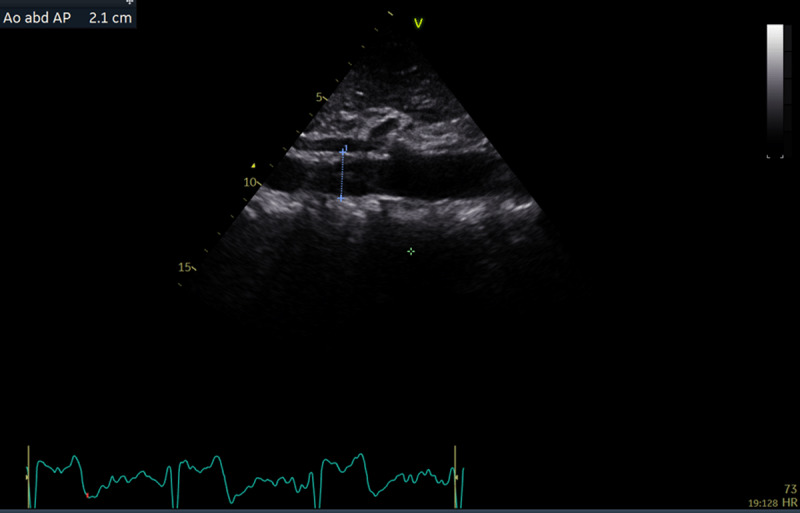
Longitudinal image of the aorta from a 49-year-old female participant. Anterior–posterior diameter (2.1 cm) is measured at the infrarenal location.

Standard TTEs were performed by using a portable ultrasound machine (Vivid, GE Healthcare). Left ventricular (LV) chamber quantification was performed according to the joint European and American guidelines (14). LV mass was calculated according to the Devereux formula and indexed for body surface area (15). Increased LV mass was defined as >95 g/m^2^ in women and >115 g/m^2^ in men when LV mass was indexed for body surface area, and LV mass as >46.7 g/m^2.7^ in women and as 49.2 g/m^2.7^ in men when LV mass was indexed for body height. Dedicated images for AA diameter measurement were obtained while the patient was in the supine position. AA diameter was measured by the leading-edge-to-leading-edge method from a longitudinal plane (16). All images were re-analysed offline in EchoPAC (GE Vingmed Ultrasound) by the same investigator (SS) for research purposes. AAA was defined as abnormal dilation of the AA diameter to 3.0 cm or greater at suprarenal or preferably infrarenal location (if visible) (Figures 2 and 3).

### Data analysis

SPSS version 29.0 (IBM corporation, Armonk, NY, USA) was used for data management and analysis. All variables were tested for normality. Categorical variables were presented as percentages or numbers and compared with the χ^2^ test. Continuous variables were presented as mean ± standard deviation (SD) and compared with the Student’s *t*-test. The prevalence of AAA was estimated as the number of AAA cases divided by the number of all screened subjects. Correlates of AA diameter were tested in univariate and multivariate linear regression analyses and reported as standardized β-coefficients and *p*-values. Multivariate models were adjusted for age, gender, smoking, atrial fibrillation, clinic systolic BP, ascending aortic diameter, aortic sclerosis (visually assessed or peak aortic jet velocity of 1.8–2.5 m/s), LV ejection fraction and LV mass. Due to a strong inverse correlation between LV end-diastolic diameter and LV ejection fraction (Pearson *R* = –0.62, *p* < 0.001), these two variables were not entered in the same multivariate model. Similarly, the left atrial volume index had a moderate positive correlation with LV end-diastolic diameter (Pearson *R* = 0.49, *p* < 0.001), LV mass (Pearson *R* = 0.48, *p* < 0.001) and atrial fibrillation (Pearson *R* = 0.53, *p* < 0.001) and an inverse correlation with LV ejection fraction (Pearson *R* = –0.43, *p* < 0.001). A *p*-value <0.05 was considered statistically significant.

## Results

In this cross-sectional study of 137 patients, 43% were men and 57% were women. The mean age was 54.4 ± 15.9 years (range 16–87 years). Table 1 shows the baseline characteristics of the total study population and in men and women. Obesity and higher clinic BP were more prevalent in women, while men were more likely to be smokers and have diabetes, coronary artery disease, atrial fibrillation and lower LV ejection fraction (Table 1). The aortic valve was bicuspid in 1 patient (0.7%) and tricuspid in 134 patients (99.3%). Although raw LV mass was higher in men than women, when adjusted for body height or body surface area, it was comparable between men and women.

**Table 1:** Baseline demographics and clinical and echocardiographic characteristics of the patients screened for abdominal aorta.


	TOTAL POPULATION (n = 137)	MEN (n = 59)	WOMEN (n = 78)	*P*

**Demographics and clinical data**				

Age (years)	54.4 ± 15.9	59.5 ± 15.7	50.6 ± 15.0	0.001

Weight (kg)	70 ± 15	68 ± 14	70 ± 16	0.440

Height (m)	1.61 ± 0.09	1.66 ± 0.08	1.57 ± 0.07	<0.001

Body mass index (kg/m^2^)	27 ± 6	25 ± 5	29 ± 6	0.001

Obesity (%)	42	27	54	0.002

Smoking (%)	9	19	1	<0.001

Clinic systolic BP (mmHg)	141 ± 23	136 ± 21	145 ± 24	0.023

Clinic diastolic BP (mmHg)	87 ± 12	84 ± 12	88 ± 12	0.036

Diabetes (%)	14	21	8	0.030

Coronary artery disease (%)	15	24	9	0.018

Atrial fibrillation (%)	13	19	9	0.097

Chronic kidney disease (%)	7	13	3	0.024

**Echocardiographic data**				

Aortic annulus diameter (cm)	2.0 ± 0.2	2.0 ± 0.3	1.7 ± 0.2	0.001

Aortic root diameter (cm)	3.1 ± 0.4	3.3 ± 0.4	2.9 ± 0.4	<0.001

Ascending aortic diameter (cm)	3.2 ± 0.4	3.3 ± 0.4	3.1 ± 0.4	0.006

Abdominal aortic diameter (cm)	2.1 ± 0.3	2.2 ± 0.3	2.1 ± 0.3	0.005

LVEF by Simpson biplane (%)	52 ± 15	49 ± 17	55 ± 14	0.027

*E/e*’ ratio	10 ± 5	10 ± 4	11 ± 5	0.406

LV mass (g)	168 ± 75	186 ± 77	155 ± 70	0.016

LV mass indexed by height (g/m^2.7^)	47 ± 20	45 ± 18	48 ± 23	0.573

LV mass indexed by BSA (g/m^2^)	97 ± 41	101 ± 39	94 ± 43	0.437

Left atrial volume index (ml/m^2^)	35 ± 18	36 ± 18	34 ± 18	0.527


BP, blood pressure; BSA, body surface area; LV, left ventricular; LVEF, left ventricular ejection fraction.

### Correlates of abdominal aortic diameter

AA could be visualized in 128 (93.4%) patients. The mean AA diameter was 2.1 ± 0.3 cm in the entire study population and was significantly greater in men than women (2.2 ± 0.3 vs 2.1 ± 0.3 cm, *p* = 0.005) and in individuals aged ≥60 years than those aged <60 years (2.3 ± 0.3 vs 2.1 ± 0.3 cm, *p* = 0.003). The prevalence of AAA was 1.6%. AA diameter had a modest but statistically highly significant correlation with the aortic root (Pearson *R* = 0.30, *p* < 0.001) and ascending aortic diameter (Pearson *R* = 0.31, *p* < 0.001). The mean AA diameter was comparable between patients with diabetes and patients without diabetes (2.2 ± 0.2 vs 2.1 ± 0.3 cm, *p* = 0.472). Univariate correlates of greater AA diameter are presented in Table 2. Smoking, diabetes, chronic kidney disease, filling pressure (*E*/*e*’ ratio), a measure of diastolic dysfunction and typical echocardiographic features of rheumatic heart disease had no association with AA diameter. In a multivariate linear regression analysis, higher age (β = 0.17, *p* = 0.048), male gender (β = 0.18, *p* = 0.036), atrial fibrillation (β = 0.22, *p* = 0.010) and LV mass (β = 0.36, *p* < 0.001) were identified as independent correlates of greater AA diameter, adjusted for smoking clinic systolic BP, ascending aortic diameter and LV ejection fraction (multiple *R^2^* = 0.38, *p* < 0.001) (Table 2). In the same primary multivariable-adjusted model, when ascending aortic diameter was replaced by aortic sclerosis, the results did not change (data not shown).

**Table 2:** Correlates of abdominal aortic diameter in univariate and multivariate linear regression analyses.


	UNIVARIATE		MULTIVARIATE	
	
	β	*P*	β	*P*

**Demographics and clinical factors**				

Age (years)	0.36	<0.001	0.17	0.048

Male gender	0.25	0.005	0.18	0.036

Height (m)	0.16	0.125		

Body mass index (kg/m^2^)	0.03	0.807		

Smoking	–0.036	0.684	–0.11	0.170

Coronary artery disease	0.12	0.165		

Atrial fibrillation	0.31	<0.001	0.22	0.010

Diabetes mellitus	0.06	0.472		

Clinic systolic BP (mmHg)	0.25	0.005	0.15	0.065

Chronic kidney disease	0.05	0.583		

**Echocardiographic parameters**				

Aortic root diameter (cm)	0.30	0.001		

Ascending aortic diameter (cm)	0.31	<0.001	–0.04	0.697

Peak aortic jet velocity (m/s)	0.19	0.032		

Aortic sclerosis	0.24	0.008	–0.04	0.958

LV end-diastolic diameter (cm)	0.289	0.001		

LVEF by Simpson biplane (%)	–0.20	0.028	–0.02	0.888

*E/e*’ ratio	0.10	0.319		

LV mass (g)	0.48	<0.001	0.36	<0.001

LV mass indexed by height (g/m^2.7^)	0.43	0.001		

LV mass indexed by BSA (g/m^2^)	0.48	<0.001		

Left atrial volume index (ml/m^2^)	0.31	<0.001		

Echocardiographic features of RHD	0.05	0.566		


BP, blood pressure; BSA, body surface area; LV, left ventricular; LVEF, left ventricular ejection fraction; RHD, rheumatic heart disease.

## Discussion

The key findings of this Zanzibar Heart Survey are (1) abdominal aorta screening during routine transthoracic echocardiograph was feasible in up to 93.4% of patients; (2) patients referred from the community for echocardiography due to cardiac symptoms had relatively smaller AA diameters compared with European population, and the prevalence of AAA was 1.6% and (3) men and individuals aged >60 years had greater AA diameters than women and individuals aged <60 years, and independent correlates of AA diameter in the entire study population were older age, male gender, atrial fibrillation and LV mass, adjusted for clinic systolic BP, ascending aortic diameter, aortic sclerosis and LV ejection fraction.

Over the past decades, Western countries have seen a significant decline in AAA prevalence and incidence rates, attributed to better risk factor control strategies including smoking cessation programmes (17). However, similar improvements in epidemiological trends with regard to AAA prevalence in the African population, especially those living in the sub-Saharan region, have not been shown or investigated. The prevalence of AAA in African countries has been reported to vary between 0.7% and 6.4% (18). However, most studies were conducted in North Africa, and data from the sub-Saharan regions were underrepresented. In a study conducted in Algeria including 600 individuals from the general population (age >60 years), the prevalence of AAA was 2.2% (19). This warranted further research based upon larger epidemiological studies to define normal values for AA diameter in African men and women and to better characterise AAA based on these values. Turkish authors have also reported a 2.2% prevalence of AAA in the general population based upon data from 5138 patients who underwent echocardiographic evaluation for AAA screening (20). Furthermore, in a study conducted in Nigeria including 400 healthy adult volunteers (mean age 51 years, 51.3% females, mean height 1.62 m and mean body mass index [BMI] 25.1 kg/m^2^), AA diameters were relatively small (mean diameter 1.58 ± 0.24 cm at the level of the superior mesenteric artery and 1.40 ± 0.19 cm at the origin of the renal arteries) and correlated with age and height (21). In our study, the mean AA diameter predominantly at the level of the superior mesenteric artery was 2.15 ± 0.32 cm. Despite a comparable body height, patients in our study were 5 years older and had a number of comorbidities and symptoms, which may explain the greater AA diameters compared with those reported in the Nigerian study (21). Moreover, we measured AA diameter by the leading-edge-to-leading-edge method, while Ezenwugo *et al*. (21) took measurements of the internal diameter from intima to intima, yielding somehow lower values. Of note, among the three commonly used ultrasound methods, leading-edge-to-leading-edge measurement has been found to be the most reproducible method for measuring the abdominal aorta diameter (16). However, the prevalence of AAA in our study was still low and the AA diameters were relatively small. A possible explanation for this may include the low population life expectancy, which is shorter in Africa (mean 63 years) (18,22), and AAA typically presents in individuals aged ≥65 years. It is important to note that our sample size was small, and the results are hypothesis-generating and should be interpreted with caution and verified in large-scale regional screening programmes.

Among the demographic and clinical factors associated with AA diameter described in the literature, we found only age and male gender as independent correlates. Smoking is an important predictor of AAA, and smoking cessation is therefore recommended to reduce the risk of progression and rupture. However, we found no association between smoking and AA diameter, probably because the prevalence of smoking was already very low in Zanzibar, as previously described, thanks to the successful campaigning of the WHO in terms of smoking cessation over the past decade (12). Besides, an independent association was found between atrial fibrillation and LV mass with AA diameter. Both atrial fibrillation and LV mass are important determinants of cardiovascular morbidity and mortality. Although aortic dilation is a phenomenon of age, and the prevalence of atrial fibrillation increases with age, the association between atrial fibrillation and AA diameter was independent of age and other well-established risk factors including systolic BP and LV mass. Factors that affect AA dilatation also lead to atrial fibrillation. Our results are in line with those reported in previous population-based cohort studies, showing an association between atrial fibrillation and AA even after adjusting for several comorbidities and medications (23).

Statins, antiplatelet therapy, beta-blockers and other antihypertensive medications are indicated to reduce cardiovascular risk but have not been shown to reduce the growth of aortic aneurysms. However, in this Zanzibar Heart Survey, LV mass but not systolic BP was found to be associated with greater AA diameter. Therefore, measures targeting reduction of LV mass through lifestyle modification including weight loss (because of the disproportionately high prevalence of obesity and hypertensive heart disease in women), optimal antihypertensive medication and optimal BP control are essential to reduce adverse cardiovascular events including stroke, aortic dissection, risk of atherothrombosis, rupture and LV dysfunction.

In a recent echocardiographic study of 307 males (199 AAA cases and 108 controls) from Sweden, the authors demonstrated that patients with AAA had larger diameters of the aortic root and ascending aorta, suggesting that AAA was a general aortic disease and not confined only to the abdominal aorta (24). However, the study included only males, and the authors acknowledged the need for more studies to investigate the correlation between AAA and cardiovascular diseases in both men and women. In our univariate analyses, we observed that LV end-diastolic diameter, aortic root and ascending aorta diameters had a positive correlation with AA diameter. However, these associations did not remain significant in the fully adjusted models.

Finally, several European studies have suggested that diabetic patients have significantly lower aortic diameters compared to non-diabetic subjects and may develop smaller AAA, with lower growth rates compared to controls (25,26,27,28). However, such a protective effect of diabetes on AA diameter was not evident in our study. We found no statistically significant association between diabetes and AA diameter, and multivariate analyses were accordingly not adjusted for diabetes. The impact of diabetes on AA diameter merits further research studies from sub-Saharan countries.

## Limitations

Our study has several limitations. First, we acknowledge the small sample size. Second, although the study provides invaluable observational insights, it was cross-sectional and not possible to infer the causality of the associations discovered in this study, as some associations may be bidirectional. However, with the data provided by our work, we have laid the foundations for further research with regard to larger screening programs for AAA in Zanzibar and other countries in the region. Third, although aneurysms can occur along the entire length of the abdominal aorta, the infrarenal aorta is the most common site for AAA development. The prevalence of AAA may be underestimated in our survey because of the difficulties with scanning the infrarenal location of the abdominal aorta with an echo probe in some patients. Finally, this was a single-centre study and referral bias could be a limiting factor.

## Conclusions

Findings from this Zanzibar Heart Survey demonstrate that rapid screening of the AA during TTE is feasible and should be part of a standard transthoracic echocardiogram. The prevalence of AAA defined as an AA diameter of ≥3 cm was 1.6%, and independent correlates of AA diameter were older age, male gender, atrial fibrillation and LV mass. AA screening by routine echocardiography may be beneficial, provided that access to care and vascular surgery facility/expertise with appropriate follow-up is available for patients with AAA identified during screening.

## Data Accessibility Statement

All data are included. There are no additional data. The data that support the findings of this survey are not publicly available due to regulatory restrictions.
